# A semi-tryptic peptide centric metaproteomic mining approach and its potential utility in capturing signatures of gut microbial proteolysis

**DOI:** 10.1186/s40168-020-00967-x

**Published:** 2021-01-12

**Authors:** Zhixiang Yan, Feixiang He, Fei Xiao, Huanhuan He, Dan Li, Li Cong, Lu Lin, Huijin Zhu, Yanyan Wu, Ru Yan, Xiaofeng Li, Hong Shan

**Affiliations:** 1 Guangdong Provincial Key Laboratory of Biomedical Imaging and Guangdong Provincial Engineering Research Center of Molecular Imaging, The Fifth Affiliated Hospital, Sun Yat-sen University, Zhuhai, 519000 Guangdong Province China; 2 Department of Endocrinology and Metabolism, The Fifth Affiliated Hospital, Sun Yat-sen University, Zhuhai, 519000 Guangdong Province China; 3 Department of Gastroenterology, The Fifth Affiliated Hospital, Sun Yat-sen University, Zhuhai, 519000 Guangdong Province China; 4 State Key Laboratory of Quality Research in Chinese Medicine, Institute of Chinese Medical Sciences, University of Macau, Taipa, Macao China; 5 Center for Interventional Medicine, The Fifth Affiliated Hospital, Sun Yat-sen University, Zhuhai, 519000 Guangdong Province China

**Keywords:** Metaproteomics, Gut microbial proteolysis, Inflammatory bowel disease

## Abstract

**Background:**

Proteolysis regulation allows gut microbes to respond rapidly to dynamic intestinal environments by fast degradation of misfolded proteins and activation of regulatory proteins. However, alterations of gut microbial proteolytic signatures under complex disease status such as inflammatory bowel disease (IBD, including Crohn’s disease (CD) and ulcerative colitis (UC)), have not been investigated. Metaproteomics holds the potential to investigate gut microbial proteolysis because semi-tryptic peptides mainly derive from endogenous proteolysis.

**Results:**

We have developed a semi-tryptic peptide centric metaproteomic mining approach to obtain a snapshot of human gut microbial proteolysis signatures. This approach employed a comprehensive meta-database, two-step multiengine database search, and datasets with high-resolution fragmentation spectra to increase the confidence of semi-tryptic peptide identification. The approach was validated by discovering altered proteolysis signatures of *Escherichia coli* heat shock response. Utilizing two published large-scale metaproteomics datasets containing 623 metaproteomes from 447 fecal and 176 mucosal luminal interface (MLI) samples from IBD patients and healthy individuals, we obtain potential signatures of altered gut microbial proteolysis at taxonomic, functional, and cleavage site motif levels. The functional alterations mainly involved microbial carbohydrate transport and metabolism, oxidative stress, cell motility, protein synthesis, and maturation. Altered microbial proteolysis signatures of CD and UC mainly occurred in terminal ileum and descending colon, respectively. Microbial proteolysis patterns exhibited low correlations with β-diversity and moderate correlations with microbial protease and chaperones levels, respectively. Human protease inhibitors and immunoglobulins were mainly negatively associated with microbial proteolysis patterns, probably because of the inhibitory effects of these host factors on gut microbial proteolysis events.

**Conclusions:**

This semi-tryptic peptide centric mining strategy offers a label-free approach to discover signatures of in vivo gut microbial proteolysis events if experimental conditions are well controlled. It can also capture in vitro proteolysis signatures to facilitate the evaluation and optimization of experimental conditions. Our findings highlight the complex and diverse proteolytic events of gut microbiome, providing a unique layer of information beyond taxonomic and proteomic abundance.

Video abstract

**Supplementary Information:**

The online version contains supplementary material available at 10.1186/s40168-020-00967-x.

## Introduction

Gut microbiota lives in a dynamic environment, facing proteotoxic and metabolic stresses from drugs, diet, microbial competitors, and host endogenous chemical components. Regulated proteolysis allows microbes to respond to stress conditions rapidly by fast and specific proteolytic degradation of misfolded or damaged proteins, activation of regulatory proteins, and production of signals [1–3]. For instance, proteolytic cleavage of a C-terminal prosequence activates leucine aminopeptidase from *Pseudomonas aeruginosa* by inducing intramolecular autocatalytic removal of a propeptide at the N terminus [4]. Proteolysis is especially important under conditions, where damaged and/or misfolded proteins are likely to accumulate, for example, at elevated temperatures or in oxidizing environments. Proteases also play an important role in multidrug tolerance with proteolytic queues at ClpXP increasing antibiotic tolerance ∼80 and ∼60 fold in an *Escherichia coli* (*E. coli*) population treated with ampicillin and ciprofloxacin, respectively [5]. In addition, it has been shown that proteolysis is essential to regulate flagellar biosynthesis [6] and remove improperly assembled spore envelopes [7] in *Bacillus subtilis*. While many studies have investigated the degradation of certain substrates in single bacterial species under simple environment stressors [3–9], no study has been performed to explore alterations of gut microbial proteolytic signatures under complex disease status.

Inflammatory bowel disease (IBD), mainly consisted of Crohn’s diseas (CD) and ulcerative colitis (UC), is a chronic inflammatory disease influenced by genetic and environmental factors. Reports have confirmed that IBD is associated with a gut microbial dysbiosis. Metagenomics and 16S rRNA gene sequencing represented the vast majority in gut microbiome researches in IBD [10–12]. However, metatranscriptomics and/or metaproteomics approaches are needed to pinpoint functional and metabolic activities by direct measuring RNAs and proteins, respectively [13–15]. Furthermore, there are important additional regulations at protein level such as controlled proteolysis that are not captured in RNA measurements but could be monitored using metaproteomics.

In a routine metaproteomics data analysis [16–19], it is necessary to select the most representative peptides to reliably quantify proteins. Usually semi-tryptic peptides are not considered because the expended search space will increase database search time and detection of semi-tryptic peptides is less consistent than that of fully tryptic peptides. In general, semi-tryptic peptides are mainly derived from endogenous proteolysis while the impacts of other factors such as in-source fragmentation and sample degradation are negligible [20, 21]. With these concepts in mind, we have developed a semi-tryptic peptide centric metaproteomic mining approach utilizing two large-scale metaproteome datasets [11, 19] and shown its potential utility in capturing signatures of altered gut proteolysis.

## Methods

### Datasets

We analyzed two published datasets of healthy and IBD gut metaproteomes. Dataset 1 (PXD008675) was comprised of 447 fecal metaproteomes from 89 subjects aged 6–58 with a median of 22.8 years, including 24 non-IBD controls, 39 individuals with CD, and 26 with UC [11]. Of these, 272 and 184 samples have matched metagenomes and metatranscriptomes, respectively. Dataset 2 (PXD007819) came from 176 mucosal luminal interface (MLI) aspirate metaproteomes collected from the ascending colon (AsC), descending colon (DeC), or terminal ileum (TI) from 71 pediatric patients (< 18 years old) including 25 CD, 22 UC, and 24 non-IBD controls [19]. We also analyzed one proteomic dataset (PXD000498) to characterize the effects of thermal stress (42 °C versus 37 °C, biological triplicates) on the proteolysis regulation of *E. coli* K-12 [22].

### Sequence database

A comprehensive human gut microbial protein database was generated by combining the following parts: (1) the integrated gene catalog (IGC) of human gut microbiome based on 1267 gut metagenomes from 1070 individuals (760 European, 368 Chinese, and 139 American samples) [23]; (2) the sequence data of 215 bacteria isolates cultured from healthy adult human feces [24]; (3) the Culturable Genome Reference (CGR) of 1520 nonredundant, high-quality draft genomes generated from > 6000 bacteria cultivated from fecal samples of healthy humans [25]; and (4) all Archaea, Bacteria, and Fungi sequences in UniProtKB (Release 2017_06) and NCBI RefSeq (Release 90). The microbial sequence database was appended by a UniProt human reference proteome (downloaded on 2017_06), a food database of dietary organisms *Triticum aestivum* (wheat), *Oryza sativa subsp. japonica* (rice), *Glycine max* (soybean), *Zea mays* (Maize), *Arachis hypogaea* (Peanut), *Solanum tuberosum* (Potato), *Solanum lycopersicum* (Tomato), *Sus scrofa* (pig), *Bos taurus* (Bovine), *Gallus gallus* (chicken), *Ovis aries* (sheep), *Salmo salar* and *Oncorhynchus mykiss* (fish), *Artemia sp.*, and *Litopenaeus vannamei* (shrimp), and a common contaminants database (http://maxquant.org/contaminants.zip). Proteins were dereplicated at 100% amino acid identity using USEARCH v11.0.667 (–fastx_uniques) [26], resulting in a total number of 130,975,891 non-redundant sequences.

### Database searching

The database searching pipeline generally included two major steps: (1) de novo sequencing and initial large database semi-tryptic search using PEAKS and (2) multiengine refined semi-tryptic search using reduced database. To handle the increased search space and time in metaproteomic semi-tryptic peptide identification, search was first performed using PEAKS DB (version X) [27] on a local 156-core cluster configured with Intel(R) Xeon(R) CPU @ 3.00GHz and 1.5 TB 2666 MHz RAM. The software first performed de novo sequencing followed by database search using the following parameters. Mass tolerance was set to 10 ppm for the precursor ion and 0.02 Da for the fragment ion. Carbamidomethylation of Cys was set as a fixed modification. The maximum number of variable posttranslational modifications per peptide was three, including acetylation of protein N-terminus, oxidation of Met, deamidation of Asn, and Gln as well as Pyro-glu from Gln. For database search, enzyme was trypsin, digest mode was semispecific, and max missed cleavages were three. The two-step strategy [19] was employed to increase the sensitivity of metaproteomics database searching. Proteins identified by at least one peptide (1% false discovery rate (FDR) using the decoy fusion approach) in the first step search were reserved for the second round multiengine database search using PEAKS DB, MaxQuant (version 1.6.2) [28], and pFind (version 3.1.5) [29].

MaxQuant (version 1.6.2.10) was performed using the Andromeda search engine [30]. Mass tolerance was set to 20 ppm for the first and 4.5 ppm for the main search. Enzyme was trypsin, digest mode was semispecific, and max missed cleavages were two. Carbamidomethylation of Cys was set as a fixed modification. The maximum number of variable posttranslational modifications per peptide was five, including acetylation of protein N-terminus, oxidation of Met, deamidation of Asn and Gln, and Pyro-glu from Gln. Peptide-to-spectrum matches, peptide, and site FDR were set to 0.01 based on the target-decoy strategy. Second peptides option was enabled to search for co-fragmented peptides in the MS/MS spectra. The “match between runs” option was enabled (without matching unidentified features) using a match time window of 0.7 min with an alignment window of 20 min. For protein quantification, a separate database searching was performed with digest mode set to specific. Protein and peptide quantification was performed using the label-free quantification (LFQ) algorithm with a minimum ratio count of 1, and minimum and average number of neighbors of 3 and 6, respectively. Reverse decoy and common contaminants matches were removed from the identification list. Peptides with local FDR (posterior error probability (PEP)) < 0.05 were kept for further analysis.

Database search using pFind was performed using a mass tolerance of 10 ppm for the precursor ion and 20 ppm for the fragment ion, respectively. Enzyme was trypsin, digest mode was semispecific, and max missed cleavages were three. The open search function [29] was selected and a 1% global FDR based on the target-decoy strategy was applied. Only peptides identified by all three searching engines were kept for further analysis.

### Semi-tryptic peptide mining

Peptides that do not have R or K (excluding protein N-terminal peptides, ~ 0.6% in our cases) in the amino acid before identified sequences were selected as semi-tryptic N-term peptides. Semi-tryptic C-term peptides were selected if the last amino acid of identified sequences lacks an R or K (excluding C terminus of the protein, ~ 2.2% in our cases). The in-source fragments were distinguished from proteolytic-derived semi-tryptic peptides based on elution time as previously reported [20]. The majority of in-source fragments showed different retention time as compared to their theoretical retention time (predicted using SSRCalc [31]) and gave the same retention time of their fully tryptic parental peptides. In addition, in-source fragments exhibit lower charge states than their corresponding parental peptides because the charge of the parental peptide is divided between fragments. Microbial semi-tryptic peptides were distinguished from human and food peptides based on the corresponding accession numbers from fasta sequence entries.

### Taxonomy and functional analysis of peptides

Analysis was performed with UniPept (version 4.3.5) [32, 33] using UniProt 2020.01 based on the lowest common ancestor (LCA) algorithm. Semi-tryptic peptides were converted to their closest fully tryptic peptides based on sequences in N-term cleavage and C-term cleavage window. All peptides were analyzed using the following parameters: equate I and L, filter duplicate peptides, and advanced missing cleavage handling. The taxonomy information was visualized using a sunburst view provided by UniPept. Functional peptide annotations were performed using Gene Ontology (GO) terms and Enzyme Commission (EC) numbers.

### Combining semi-tryptic and fully tryptic peptide data to quantify the degree of proteolysis

We determined alterations in the degree of proteolysis based on the normalized relative abundance of semi-tryptic peptides (NRASP) by normalizing semi-tryptic peptide-based relative abundance to fully tryptic peptide-based relative abundance. The logic is similar to that employed in other post-translational modification (PTM) studies, where PTM occupancy determination is achieved by measuring the abundances of both PTM and non-PTM peptides.

### Nomenclature and sequence motif of cleavage sites

According to the nomenclature of Schechter and Berger [34], amino acids around the cleavage sites were numbered as P6-P5-P4-P3-P2-P1 ↓ P1′-P2′-P3′-P4′-P5′-P6′, with the downward-pointing arrow indicating the cleaved peptide bonds between the P1 and P1′ sites. We retrieved amino acids in P6-P6′ of each semi-tryptic peptide from MaxQuant outputs (N-term cleavage window and C-term cleavage window of the peptides.txt file) using Microsoft Excel formulas and functions. The relative frequency of each amino acid at P6-P6′ was calculated to determine the inter-group differences. To visualize conserved and frequently occurring amino acids at positions flanking the cleavage site, sequence motif logos were generated using Weblogo [35]. Each logo consists of stacks of symbols; one stack for each position in the sequence. The height of symbols within the stack indicates the relative frequency of each amino acid at that position.

### Statistical analysis

Multivariate analyses of the amino acid frequencies around the cleavage sites were performed using principal component analysis (PCA) and partial least squares discriminant analysis (PLS-DA) with missing value imputed by Bayesian PCA (BPCA) [36]. Dunn-Bonferroni post hoc procedure following Kruskal-Wallis test with a threshold of adjusted *P* value < 0.05 was employed to detect significantly different variables (present in at least 75% of samples) among groups using R (vesion 3.5.3) and RStudio (version 1.1.383). Beta-diversity of multi-omics data were determined using principal coordinate analysis (PCoA) using the Bray-Curtis distance [37]. The correlation between microbial semi-tryptic peptide intensity and multi-omics data was evaluated by Spearman rank correlation of their top three PCos (PCo1-PCo3).

## Results

### A pipeline for metaproteomic semi-tryptic peptide characterization

#### High-confidence identification of semi-tryptic peptides

**Fig. 1:**
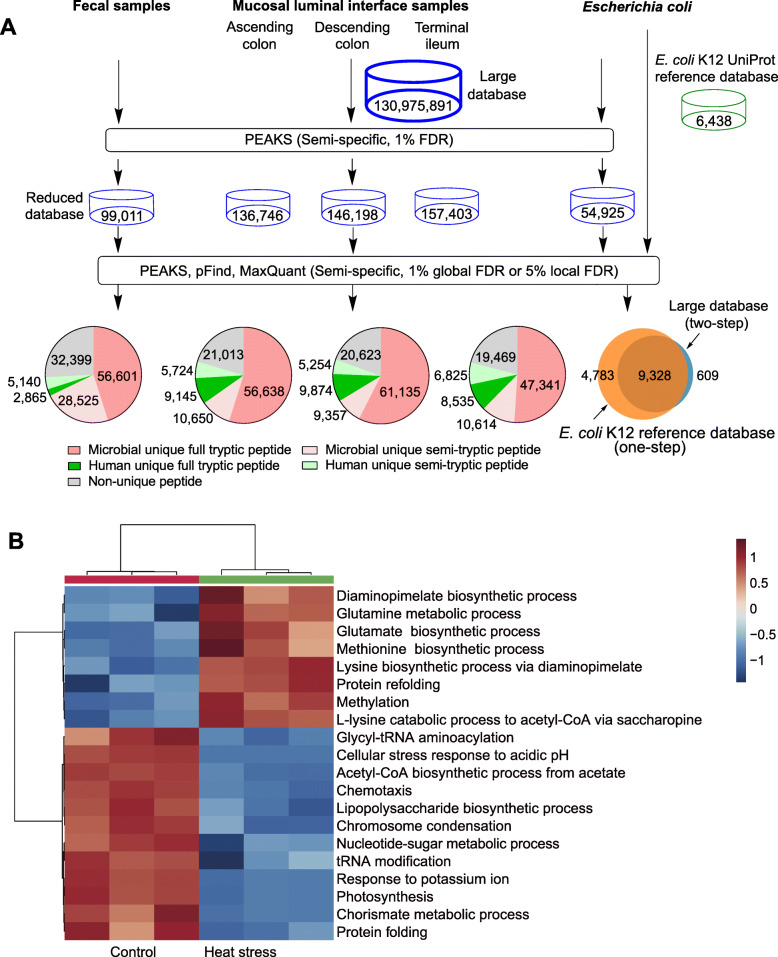
Workflow and validation of semi-tryptic peptide centric metaproteomic mining approach. The approach was applied to analyze the fecal and MLI metapeoteomes and validated using the *E. coli* heat-shock-induced proteome (**a**). Alerted proteolysis signatures of different biological processes induced by heat stress in *E. coli* proteome (**b**, adjusted *P* < 0.05)

Using two large-scale published datasets including 447 fecal (PXD008675, [11]) and 176 MLI metaproteomes (PXD007819, [19]), we have developed a pipeline (Fig. 1a) for efficient and high-confident metaproteomic characterization of semi-tryptic peptides which represented potential proteolytic signatures of gut microbiome. Both datasets were generated using high-resolution MS/MS, which allowed searching a large sequence space with a low FDR for semi-tryptic peptide identification.

The first critical step in metaproteome mining is the database construction. Several metaproteomics studies have employed costly and time-consuming sample-specific protein database by metagenomic sequencing of each sample [11, 17]. Metagenome-matched database may also suffer from technical issues in DNA extraction and bioinformatics issues, making cross-study comparisons difficult [38]. Furthermore, sample aliquots used in metagenomic sequencing may not be exactly the same as those used in metaproteomics due to sample heterogeneity. Therefore, we assembled microbial sequences from public repository including a variety of culture-dependent sources such as UniProtKB, NCBI, CGR [25], and culture-independent sources such as IGC [23] to increase microbial taxonomic coverage and facilitate cross-study comparisons. The combination of microbial sequences with human sequence and a comprehensive food database of most common dietary organisms resulted in a total number of 130,975,891 non-redundant sequences. Using the MLI dataset, we compared different commercial software (Proteome Discoverer, PEAKS, ProteinPilot, and Byonic) and open-source packages (MaxQuant, MSFragger, and pFind) in their performance of large database semi-tryptic search on several 36-core servers (192G RAM installed). Proteome Discoverer, Byonic, MaxQuant, pFind, and ProteinPilot (stuck on the “progroup” step for more than 2 weeks) did not complete the search in 1 month, and MSFragger crashed with an out of memory error. Only PEAKS completed the analysis in 1 month and was selected for further high-throughput analysis using a 156-core cluster which completed database search in 2 weeks. Other ultrafast metaproteomics search engines such as ComPIL [39] and ProteoStorm [40] may also be used in the first step large database search.

A total number of 12,828,005, 3,133,023, 2,948,562, and 2,757,990 MS/MS spectra were searched for the feces, ascending colon (AsC), descending colon (DeC), and terminal ileum (TI) MLI metaproteomes, respectively, from which 3,804,903 (29.66%), 2,035,847 (64.98%), 1,917,761 (65.04%), and 1,808,732 (65.58%) peptide-spectrum matches (PSMs) were identified using PEAKS (1% FDR) in the first-step large database search. To increase the sensitivity of large sequence space-based metaproteomic analysis, we employed the two-step database searching strategy [18]. This facilitated the semi-tryptic-based metaproteomics search by reducing the size of database to that of conventional proteomics analysis (99,011 sequences for feca metaprotelome, 136,746 for AsC, 146,198 for DeC, and 157,403 for TI MLI metaproteomes, respectively). Furthermore, we applied stringent criteria to increase the peptide identification confidence by combing three commonly used software packages. Proteins identified with at least one peptide in the first step were reserved for the second round search using MaxQuant, PEAKS, and pFind. We only considered peptides that were simultaneously identified by MaxQuant (5% local FDR), PEAKS DB (1% global FDR), and pFind (1% global FDR). These packages use different algorisms to do peak detection, cofragmented peptide identification, and FDR calculation (MaxQuant and pFind using the target-decoy strategy while PEASK DB using the decoy fusion approach), thus significantly increasing the confidence of peptide identifications. Specifically, we selected PEAKS, pFind, and MaxQuant, to increase the confidence of semi-tryptic peptide identification because these engines are featured with de novo sequencing, open-search, and match-between-runs functions, respectively. De novo sequencing was employed to reduce false positive identifications because peptides absent in sequence database can be misassigned to a sequence present in the database. Open-search was used to reduce false positive identifications because modified peptides can be misassigned to wrong sequences when modifications are not considered in conventional database search [41]. Finally, a cross-assignment procedure (known as “match between runs” in MaxQuant) was applied to recover MS1 signals missed by MS/MS.

Only peptides identified by all three software were kept for further analysis, resulting in 125,494, 103,170, 106,243, and 92,784 peptides identified in the fecal, AsC, DeC, and TI metaproteomes, respectively (Additional file 1: Tables S1-S16), among which 108,784 (86.68%), 76,325 (73.97%), 77,341 (72.79 %), and 65,002 (70.06%) peptides were assigned as microbial unique peptides (not shared by human or food sequences, Fig. 1a). Using UniPept, a total of 85,126 (78.25%), 67,288 (88.16%), 70492 (91.14%), and 57955 (89.16%) microbial peptides could get taxonomic and/or functional annotations in the fecal, AsC, DeC, and TI metaproteomes, respectively. Despite of the comprehensiveness of IGC, which was frequently employed in previous metaproteomics studies, 11,540 (10.61%), 9025 (11.82%), 9129 (11.80%), and 7308 (11.24% TI) microbial peptides were only captured by UniProt/NCBI/CGR in the fecal, AsC, DeC, and TI metaproteomes, respectively. This was probably because the UniProt/NCBI/CGR database is largely based on the translation of a completely sequenced single microorganism genome, the depth, and assembly quality of which are significantly increased compared with that of gut microbial metagenomes. Among all identified microbial peptides, 28,525 (26.22%), 10,650 (13.95%), 9357 (12.10%), and 10,614 (16.33%) peptides were semi-tryptic in the fecal, AsC, DeC, and TI metaproteomes, respectively (Additional file 1: Tables S1 and S3-S5). Although we used the “match between runs” of option MaxQaunt to increase transferred identification between separate LC-MS runs, the percentage of semi-tryptic peptides identified in more than 75% of samples was less than 0.05%. We did not considered peptides non-tryptic peptides because our initial non-enzymatic search revealed that they generally accounted for less than 0.2% of total identified peptides. However, a non-enzymatic search significantly increased the search time by several times compared with a semi-tryptic search for MaxQuant.

We identified 7969 (6.35%), 14,869 (19.48%), 15,128 (14.24%), and 15,360 (16.55%) human-specific peptides in the fecal, AsC, DeC, and TI metaproteomes, respectively, among which 5104 (64.05%), 5724 (38.50%), 5254 (34.73%), and 6825 (44.43%) peptides were semi-tryptic (Additional file 1: Tables S2 and S6-S8). Gene ontology (GO) analysis revealed that 84.13%, 79.97%, 81.74%, and 80.18% of human semi-tryptic peptides were derived from potential extracellular proteins while only 1.16%, 0.80%, 0.73%, and 0.76% of microbial semi-tryptic peptides were assigned to potential extracellular proteins in the fecal, AsC, DeC, and TI metaproteomes, respectively. The higher percentage of extracellular proteins, which are more susceptible to the gut luminal and mucosal proteases, contributed to the higher proportion of semi-tryptic peptides for human proteins.

Because many food resource such as pig, bovine, and other mammals share a large number of sequences with humans, the number of food unique semi-tryptic peptides was negligible, generally below 50 per sample after excluding peptides shared by humans (thus was not considered for further analysis). In addition, food proteins could be extensively hydrolyzed by gastric pepsin, pancreatic proteases, and small intestinal exopeptidases before they reach the large intestine (colon). So food-derived semi-tryptic peptides represent complex proteolytic events across the entire digestive system.

#### Relative abundance and distribution of semi-tryptic peptides

We first calculated the normalized relative abundance of semi-tryptic peptides (NRASP) of different microbial and functional groups by normalizing semi-tryptic peptide-based relative abundance to fully tryptic peptide-based relative abundance to determine the relative degree of proteolysis. This normalization step is important because if the abundance of semi-tryptic peptides and fully tryptic peptides changed proportionally, it generally indicates no change in the degree of proteolysis. In such cases, however, if only semi-tryptic peptides were compared, there will be inter-group difference.

**Fig. 2:**
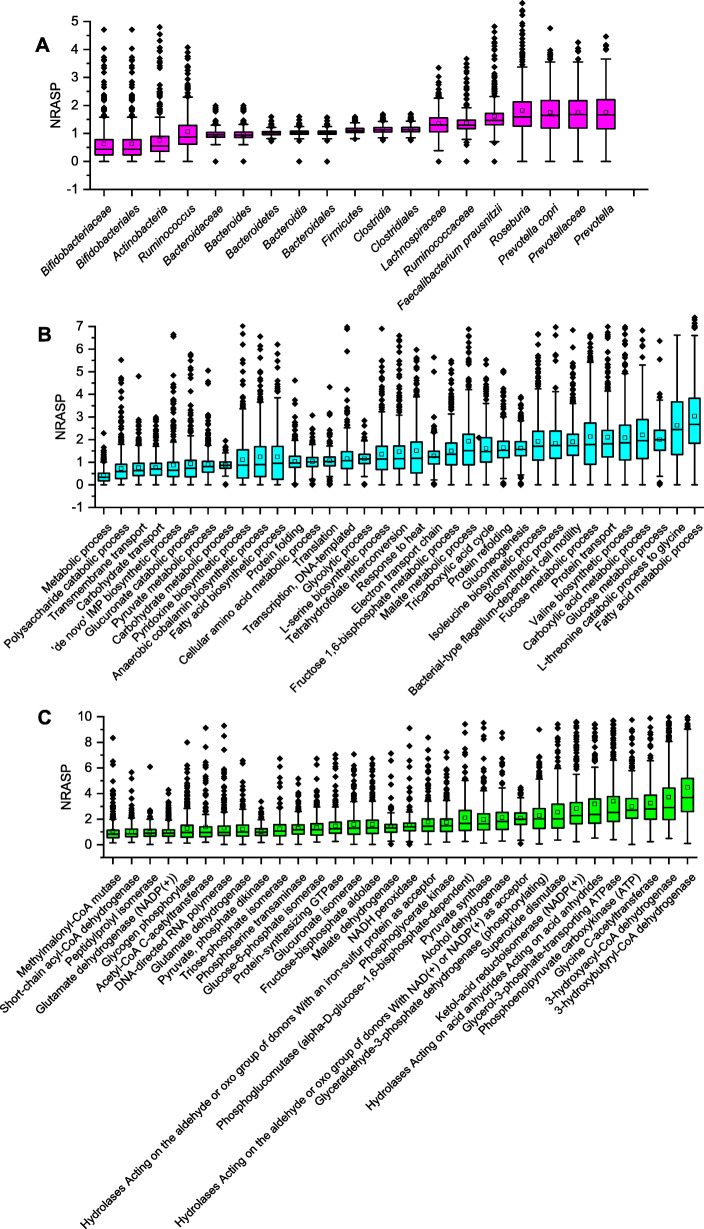
Normalized relative abundance of semi-tryptic peptides (NRASP, semi-tryptic peptide abundance/fully tryptic peptide abundance) derived from major microbial groups and biological processes in 447 fecal metaproteomics samples. Features are ranked in ascending order for different groups of bacteria (**a**), biological processes (**b**), and enzymes (**c**). Box plots represent the median (the line in the middle of the box), 25th and 75th percentiles, whiskers represent 1.5 times the interquartile range (IQR), and outliers are shown as dots

Figure 2 illustrated the overall NRASP distribution of 20 major taxonomic sub-groups, 35 major biological processes, and 32 enzyme sub-classes (Additional file 1: Tables S17-S19) identified in at least 75% of the 447 fecal metaproteomes. The median values of NRASP of two dominant phyla *Firmicutes* and *Bacteroidetes*, two dominant classes *Bacteroidia* and *Clostridia*, two major orders *Bacteroidales* and *Clostridiales*, family *Bacteroidaceae*, and genus *Bacteroides* were around 1 with a very low individual variation, suggesting the relative abundance of the corresponding semi-tryptic peptides was comparable to that of fully tryptic peptides (Fig. 2a). However, the median of NRASP increased to approximately 1.25 for families *Lachnospiraceae* and *Ruminococcaceae*, and 1.5 for genera *Roseburia* and *Prevotella* as well as two abundant species *Faecalibacterium prausnitzii* and *Prevotella copri*, respectively. In contrast, the median of NRASP reduced to approximately 0.5 for phylum *Actinobacteria* and order *Bifidobacteriales* (including its single family member, *Bifidobacteriaceae*).

The median of NRASP of most biological processes also fluctuated around 1 (Fig. 2b). However, these values increased to 1.75–2 for isoleucine biosynthetic process, valine biosynthetic processes, bacterial-type flagellum-dependent cell motility, protein transport, carboxylic acid metabolic process, fucose metabolic process, and glucose metabolic process and further increased to 2.5 for fatty acid metabolic process and L-threonine catabolic process to glycine, but reduced to approximately 0.75 for polysaccharide catabolic process, carbohydrate transport, and transmembrane transport and further reduced to 0.3 for metabolic process, respectively.

At enzyme level, 3-hydroxybutyryl-CoA dehydrogenase, which is involved in butyrate metabolism, showed the highest NRASP (median value > 3), followed by 3-hydroxyacyl-CoA dehydrogenase involved in fatty acid beta-oxidation, glycine C-acetyltransferase involved in L-threonine degradation, phosphoenolpyruvate carboxykinase (ATP) involved in gluconeogenesis, ketol-acid reductoisomerase (NADP(+)) involved in the biosynthesis of branched-chain amino acids (BCAA), and superoxide dismutase involved in tolerance to oxidant stress (median NRASP of 2–3, Fig. 2c).

#### Protease cleavage motif

**Fig. 3:**
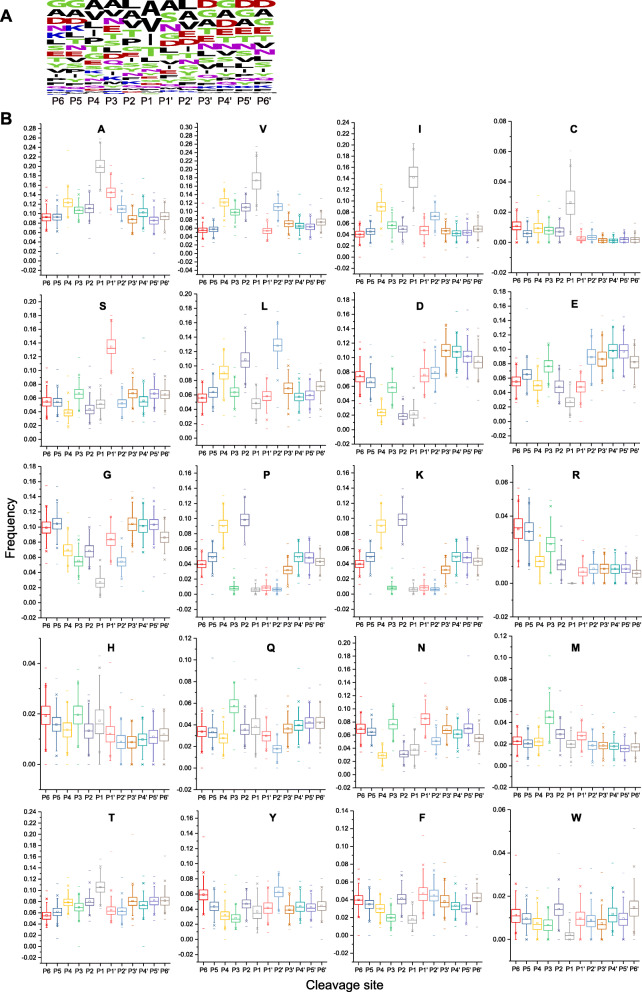
Amino acid conservation and frequency at P6-P6′ position of cleaved microbial proteins in 447 fecal metaproteomics samples. **a** A representative WebLogo of microbial proteolytic cleavage motif (P6-P6′) in a fecal metaproteome. The height of symbols within the stack indicates the relative frequency of each amino acid at that position. **b** Amino acid frequency at P6-P6′ position

We further investigated the microbial proteolytic cleavage site motif by calculating the amino acid frequencies at P6-P6′ position based on semi-tryptic peptides of 447 fecal metaproteomes (Fig. 3). Generally, alanine and valine were the most abundant amino acids at P1 position in different samples (Fig. 3a). Alanine, valine, isoleucine, and cysteine were significantly enriched at P1; serine was enriched at P1′; and leucine was enriched at P2 and P2′. Glycine was significantly reduced at P1, and proline was reduced at P1, P3, P1′, and P2′ (Fig. 3b). Two acidic amino acids (aspartic acid and glutamic acid), which were enriched in P3′-P6′, exhibited similar distribution pattern across P6-P6′. The pattern of two basic amino acids (lysine and arginine) also resembled each other with a higher frequency at P5 and P6.

### Validation of the approach by analyzing proteolysis signatures in *E. coli* heat-shock response

We validated our approach by analyzing the heat-shock-induced proteolysis signatures using a published proteomic dataset of *E. coli* K12 [22], for which proteolytic regulation rules have been increasingly reported. Combining three search engines, we identified 9937 peptides using the large database and 14111 peptides using UniProt *E. coli* K12 reference database, respectively (Additional file 1: Tables S20 and S21). The 29.6% decrease in identified peptides between two methods reflects an expected sensitivity loss since the large database produces more than 10,000-fold more sequences than conventional reference sequence. Among 4783 peptides only identified by *E. coli* reference database, 60.3% have a PEP value below 0.01 and 39.5% have a PEP value below 0.001. In contrast, among all 14111 peptides identified using *E. coli* reference database, 83.7% have a PEP value below 0.01, and 61.6% have a PEP value below 0.001. The fact that peptides only identified by *E. coli* reference database have higher PEP values demonstrates that lower quality PSMs are more susceptible to the sensitivity reduction using large database search. Meanwhile, it should be noted that the single microorganism proteome is significantly different from gut metaproteome. A recent study has shown that large public database and sample-matched reference database generated comparable results for gut metaproteomics research [18]. So our approach should not suffer from high sensitivity loss in gut metaproteome analysis. Importantly, 9328 (93.4% of 9937) peptides were identified by both methods (Fig. 1a). Manual inspection of 609 peptides that were only identified by the large database revealed that the majority of these peptides were identified with high confidence (e.g., well-annotated MS/MS spectra with high-peptide sequence coverage) and thus should not be incorrect identifications. These peptides could be assigned to *E. coli* closely related bacteria species such as *Escherichia albertii* or *E. coli* sequences deposited in NCBI (absent in UniProt). It is not surprising that the UniProt *E. coli* K12 reference database did not contain all the sequences of proteins present in the samples due to the high mutation frequency and genetic polymorphism of bacteria. Taken together, these results indicate the high-peptide identification accuracy of our approach.

To validate the biological findings of our approach, we compared the NRASP (as an indicator of proteolysis regulation) of 185 biological processes detected in all samples and found NRASP of 20 (10.8%) biological processes was significantly different between control and heat stress groups (FDR adjusted *P* value < 0.05, Fig. 1b and Additional file 1: Tables S22). Heat stress perturbs protein folding, leading to the accumulation of misfolded proteins which need to be refolded into the correct conformation. Accordingly, we found NRASP of protein refolding increased while that of protein folding reduced under heat stress. We observed NRASP of methylation increased in response to heat stress, which was in accordance with the recent finding that certain adenosines within the 5′UTR of newly transcribed mRNAs are preferentially methylated under heat shock condition [42]. Additionally, NRASP of biosynthetic processes of glutamine, methionine, and lysine also increased in heat stress group. It has been demonstrated that glutamine synthesis could maximize heat shock protein expression in Drosophila Kc cells [43]. Interestingly, we found NRASP of acetyl-CoA biosynthetic process from acetate increased but that of L-lysine catabolic process to acetyl-CoA via saccharopine increased under heat stress. Overall, our approach confirms previous findings and could provide new insights into microbial proteolysis regulation.

### Semi-tryptic peptide association with microbial composition, proteases, and chaperones

**Fig 4:**
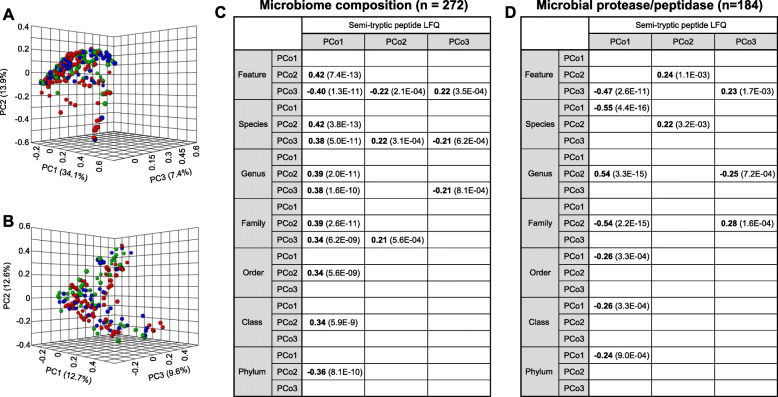
Correlation analysis between gut microbial proteolysis pattern, composition, and protease expression. Principal coordinates analysis (PCoA) of metagenome (**a**) and microbial protease/peptidase transcriptome (**b**) is based on Bray-Curtis distance. Correlations between microbial proteolytic signatures (PCo1-PCo3 of semi-tryptic peptide LFQ), microbiome composition (*n* = 272, **c**), and protease/peptidase expression (*n* = 184, **d**) were calculated using Spearman’s rank correlation based on PCo1-PCo3 of metagenome and metatranscriptome, respectively. Only significant correlations with coefficient > 0.2 or < −0.2 (*P* < 0.05) are shown in **c** and **d**. The *P* value for each correlated pair is in parentheses

To explore the potential relevance of gut microbial community structure to semi-tryptic peptide patterns, we associated the top three principal coordinates (PCo1-PCo3) of fecal microbial semi-tryptic peptide LFQ intensity and the top three microbiome β-diversity principal coordinates (PCo1-PCo3, Fig. 4a) computed using Bray-Curtis distance. We found low correlations between semi-tryptic peptide LFQ intensity (PCo1) and β-diversity (PCo2 and PCo3) at different taxonomic levels (-0.40 < Spearman’s rank correlation coefficient (*R*) < 0.42, *P* < 5.6e−9 for all pairwise associations, *n* = 272, Fig. 4c). To investigate the association with microbial proteases, we resorted to the transcriptional abundance of microbial protease/peptidase (Fig. 4b) because protein-level abundance was inaccessible due to the limited sensitivity of current metaproteomics methodology. The microbial protease/peptidase transcriptome at feature (PCo3), species (PCo1), genus (PCo2), and family (PCo2) levels showed moderate correlations with semi-tryptic peptide LFQ intensity (PCo1), stronger than those of β-diversity (Fig. 4d, − 0.55 < *R* < 0.54, *P* < 2.6e−11, *n* = 184). However, there were only low correlations at higher taxonomic levels (order, class, and phylum). In regulating cellular processes, chaperones and proteases both respond to protein misfolding and play important roles in protein homeostasis. The protein levels of chaperones could be directly measured by metaproteomics because of their high abundance. We observed that the LFQ intensities of chaperones DnaK, GroEL, ClpB, and HtpG of *Bacteroides* (the dominant species of phylum *Bacteroidetes* in human gut microbiome) as well as DnaK and GroEL of *Faecalibacterium* (the dominant species of phylum *Firmicutes* in human gut microbiome) were moderately correlated with the PCo1 of semi-tryptic peptide LFQ patterns (0.58 < *R* < 0.69, *P* < 5.0e−10, *n* = 447).

### Semi-tryptic peptide association with host protease inhibitors and immunoglobulins

In addition to microbial variables, we also investigated the involvement of host factors. Human endogenous protease inhibitors are particularly present in the intestinal tract. Four human protease inhibitors (serpin A1, A3, B1, and B6) were identified in fecal metaproteomes. Serpin A1, A3, and B6 exhibited negative correlations (− 0.41 < *R* < − 0.25, *P* < 7.9e−8, *n* = 447) with the semi-tryptic peptide LFQ intensity (PCo1 and PCo2). To further investigate the effect of host factors on gut microbial proteolysis, we also analyzed the correlations between gut microbial proteolysis pattern and host immunoglobulins. IgG1, IgG4, and IgM were negatively correlated with PCo1 (− 0.44 < *R* < − 0.25, *P* < 8.4e−8, *n* = 447), and IgA was positively correlated with PCo2 (*R* = 0.33, *P* < 8.4e−13, *n* = 447) of semi tryptic peptide LFQ intensity, respectively.

### Semi-tryptic peptide analysis reveals potential signatures of altered microbial proteolysis

**Fig. 5:**
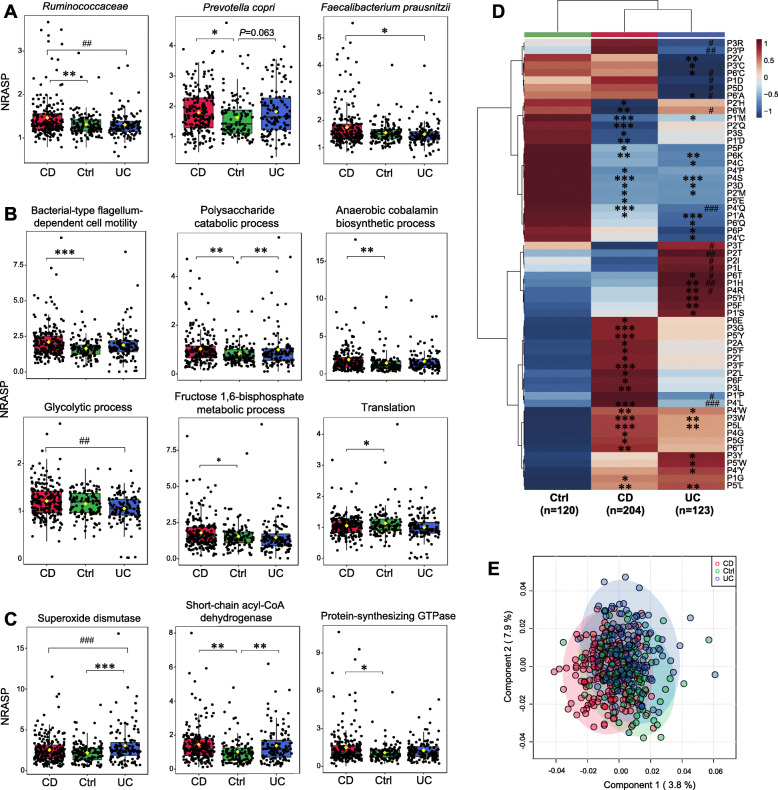
Signatures of altered microbial proteolysis at different levels in the fecal metaproteome of IBD. Normalized relative abundance of semi-tryptic peptides (NRASP) reveals altered microbial proteolysis at different taxonomic levels (**a**) as well as in different biological processes (**b**) and enzyme sub-classes (**c**). **d** Hierarchical clustering analysis of altered amino acid frequencies around the cleavage sites of microbial proteins in IBD. Partial least squares discriminant analysis (PLS-DA, **e**) of amino acid frequencies around the cleavage sites. Dunn-Bonferroni post hoc analysis following Kruskal-Wallis test was employed to detect significant difference among three groups (CD, Ctrl, and UC). *P < 0.05 versus Ctrl; **P < 0.01 versus Ctrl; ***P < 0.001 versus Ctrl; ^#^*P* < 0.05 (CD versus UC); ^##^*P* < 0.01 (CD versus UC); ^###^*P* < 0.001 (CD versus UC)

**Fig. 6:**
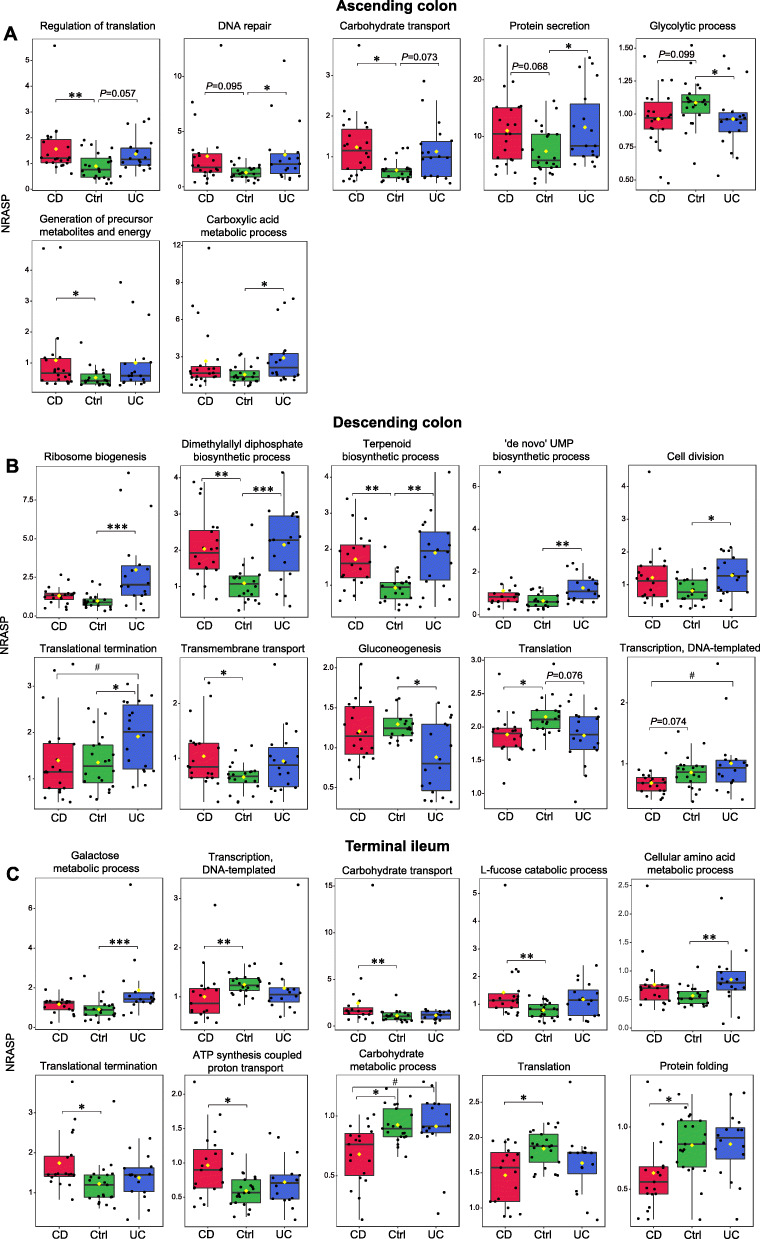
Signatures of altered microbial proteolysis at different intestinal locations in the mucosa-luminal interface metaproteome of IBD based on normalized relative abundance of semi-tryptic peptides (NRASP). **a** Ascending colon, **b** descending colon, and **c** terminal ileum. Dunn-Bonferroni post hoc analysis following Kruskal-Wallis test was employed to detect significant difference among three groups (CD, Ctrl, and UC). *P < 0.05 versus Ctrl; **P < 0.01 versus Ctrl; ***P < 0.001 versus Ctrl; ^#^*P* < 0.05 (CD versus UC); ^##^*P* < 0.01 (CD versus UC); ^###^*P* < 0.001 (CD versus UC)

**Fig. 7:**
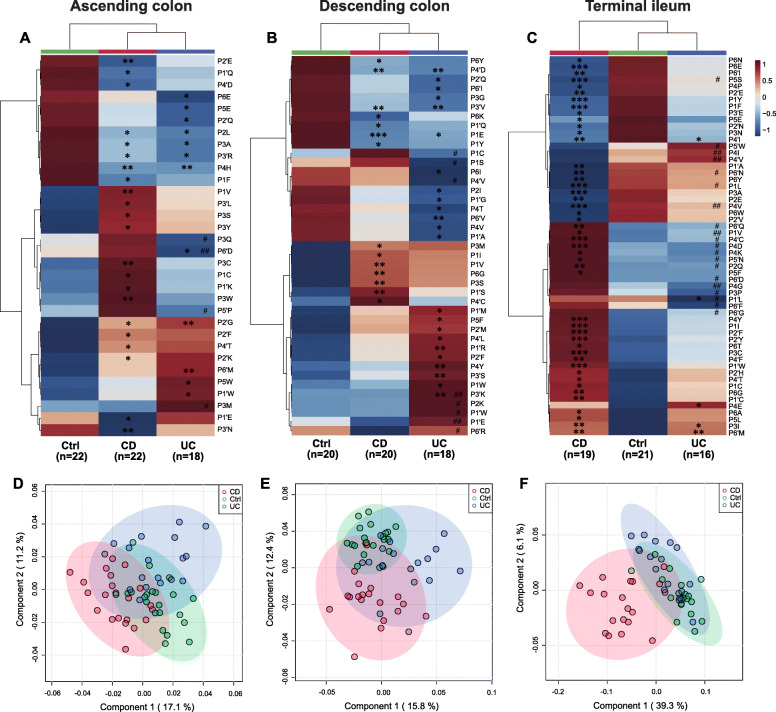
Location-specific alterations of gut microbial proteolytic motif in the MLI metaproteome of IBD. **a** and **d** ascending colon, **b** and **e** descending colon, and **c** and **f** terminal ileum. Hierarchical clustering analysis (**a–c**) and partial least squares discriminant analysis (PLS-DA, **d–f**) of altered amino acid frequencies around the cleavage site in IBD. Group averages are shown in the heatmap. Dunn-Bonferroni post hoc analysis following Kruskal-Wallis test was employed to detect significant difference among three groups (CD, Ctrl, and UC). *P < 0.05 versus Ctrl; **P < 0.01 versus Ctrl; ***P < 0.001 versus Ctrl; ^#^*P* < 0.05 (CD versus UC); ^##^*P* < 0.01 (CD versus UC); ^###^*P* < 0.001 (CD versus UC)

Using NRASP as an index of relative degree of proteolysis, we found significant inter-group differences in terms of the taxonomic and functional distributions as well as the cleavage motif in both the fecal (Fig. 5) and MLI (Figs. 6 and 7) metaproteomes. In the 447 fecal metaproteomes (including 204 CD, 123 UC, and 120 control samples), four out of 20 major taxonomic sub-groups exhibited significant inter-group difference (Kruskal-Wallis and Dunn-Bonferroni test, *P* < 0.05) (Additional file 1: Tables S17): family *Ruminococcaceae* and species *Prevotella copri* in CD have increased NRASP compared with the control group; genus *Faecalibacterium* and species *Faecalibacterium prausnitzii* in UC have reduced NRASP compared with the CD group (Fig. 5a). Six out of 35 major biological processes exhibited significant inter-group difference (*P* < 0.05) (Additional file 1: Tables S18): bacterial-type flagellum-dependent cell motility, polysaccharide catabolic process, anaerobic cobalamin biosynthetic process, and fructose 1,6-bisphosphate metabolic process in CD have increased NRASP compared with the control group; translation and glycolytic process in UC have reduced NRASP compared with the control and CD group (Fig. 5b). Four out of 32 major enzyme sub-classes exhibited inter-group difference (Additional file 1: Table S19): superoxide dismutase in UC, protein-synthesizing GTPase in CD, and short-chain acyl-CoA dehydrogenase in both CD and UC have increased NRASP compared with the control group (Fig. 5c).

In contrast, using full tryptic peptide-based relative abundance as performed in conventional metaproteomics workflow, we identified 81 major taxonomic sub-groups, 153 major biological processes, and 195 major enzymes (present in at least 75% of the samples), 4-6-fold higher than their semi-tryptic peptide counterparts. This highlights that a large number of proteolysis signatures were obscured because of the limited sensitivity of current analytical platform and bioinformatics workflow of metaproteomics. Using full-tryptic peptide-based analysis, 34 taxonomical groups, 63 biological processes, and 87 enzyme groups exhibited significant inter-group differences (Kruskal-Wallis and Dunn-Bonferroni test, *P* < 0.05) (Additional file 1: Tables S23-S25). In most cases, the taxonomic and functional alterations revealed by full tryptic peptides did not overlap with those calculated by NRASP. For instance, the relative abundance of phylum *Firmicutes and* genera *Ruminococcus* and *Alistipes* significantly decreased in IBD but their NRASP did not differ between groups (Additional file 1: Fig. S1a). The relative abundance of proteins involved in glucuronate catabolic process and glutamate metabolic process increased in IBD while those involved in nitrogen compound metabolic process and anaerobic respiration decreased in IBD (Additional file 1: Fig. S1b). However, none of these biological processes exhibited significant inter-group differences in the NRASP. NRASP of superoxide dismutase significantly increased in UC (Fig. 5c), but the relative abundance of this enzyme did not differ between groups (Additional file 1: Fig. S1c). We also observed that some taxonomic and functional alterations were identified by both NRASP and full-tryptic peptide-based comparison, such as taxonomic groups of family *Ruminococcaceae* and species *Prevotella copri*, biological processes of bacterial-type flagellum-dependent cell motility and polysaccharide catabolic process, as well as enzyme short-chain acyl-CoA dehydrogenase (Fig. 5a–c and Additional file 1: Fig. S1). Taken together, the semi-tryptic peptide centric mining approach captures a different layer of information obscured in conventional metaproteomics workflow.

The fecal metaproteome also revealed a global alteration of microbial proteolytic motif (Fig. 5d and Additional file 1: Table S26). In the unsupervised hierarchical clustering, CD and UC clustered together (separated from control), sharing many alterations such as increased frequencies of leucine (P5 and P5′) and tryptophan (P3 and P4′) as well as decreased frequencies of alanine (P1′), methionine (P1′ and P2′), aspartic acid (P3), and serine (P4). However, the large inter-individual variation did not allow for clear separation between groups in the partial least squares discriminant analysis (PLS-DA, Fig. 5e) of cleavage motif. We also performed principal coordinate analysis (PCoA) of semi-tryptic peptide LFQ abundance using Bray-Curtis distance or Jaccard-based dissimilarity but did not result in clear group separation either (Additional file 1: Fig. S2). Altered cleavage motifs of human proteins were also observed (Additional file 1: Fig. S3 and Table S27). Similar to microbial cleavage motif, CD and UC clustered together and separated from control for human protein cleavage motif. Microbial proteins and human proteins can exhibit similar or reversed alteration trends in certain motif positions around the cleavage site in IBD (Additional file 1: Fig. S4). For instance, frequencies of glutamic acid, histidine, and three aromatic amino acids (phenylalanine, tryptophan, and tyrosine) were increased in P5′ in IBD for both microbial and human proteins.

A total of 57, 58, and 51 biological processes were identified in at least 75% of samples in AsC, DeC, and TI MLI metaproteome, respectively, among which 7 (12.28%), 10 (17.86%), and 10 (19.61%) biological processes exhibited significant inter-group difference in their NRASP (Additional file 1: Tables S28-S30). In the AsC metaproteomes, NRASP of regulation of translation, carbohydrate transport, DNA repair, protein secretion, generation of precursor metabolites and energy, and carboxylic acid metabolic process significantly increased in CD and/or UC (Fig. 6a). In the DeC metaproteomes, most alterations occurred in UC, including increased NRASP of ribosome biogenesis, terpenoid biosynthetic process, “de novo” UMP biosynthetic process, cell division, and translational termination, as well as reduced NRASP of gluconeogenesis (Fig. 6b). In contrast, in the TI metaproteomes, most alterations occurred in CD, including increased NRASP of carbohydrate transport, L-fucose catabolic process, translational termination, and ATP synthesis-coupled proton transport as well as reduced NRASP of transcription, translation, protein folding, and carbohydrate metabolic process (Fig. 6c). The microbial cleavage motif also revealed remarkable location-specific alterations in MLI metaproteomes (Fig. 7a–c). Similar to NRASP, microbial cleavage motif differed more in DeC and TI than AsC, where 41, 57, and 32 amino acid frequencies at a specific site exhibited significant inter-group differences, respectively (*P* < 0.05, Additional file 1: Tables S31-S33). In the unsupervised hierarchical clustering, CD and UC clustered together, separated from control in the ascending colon (Fig. 7a), whereas UC and control clustered together and separated from CD in the terminal ileum (Fig. 7c). The supervised PLS-DA also revealed that, on PC1 axis, UC partially separated from CD and control in the descending colon and CD clearly separated from the other two groups in the terminal ileum (Fig. 7d–f). Altered cleavage motif of human proteins was also observed in the MLI metaproteomes (Additional file 1: Fig. S5 and Tables S34-S36). Similar to cleavage motif of microbial proteins, cleavage motif of human proteins in CD separated from the other two groups in the terminal ileum. Microbial proteins and human proteins can exhibit similar or reversed alteration trends in certain motif positions in IBD (Additional file 1: Fig. S6). For instance, increased frequencies of valine and leucine (P1) and reduced frequencies of two aromatic amino acid phenylalanine and tyrosine (P1) in IBD were observed in both microbial and human motif in MLI metaproteomes from different locations.

## Discussion

Our approach incorporates two-step search, de novo sequencing, open-search, and match between runs to perform large-scale semi-tryptic peptide centric metaproteomic mining. These strategies could reduce false positive identifications derived from incomplete database and peptide modifications. A preliminary study has performed semi-tryptic search of several metaproteomics samples using datasets generated by low-resolution MS/MS [44], which inevitably increased the search space and reduced the identification confidence. In their study, only 80.2% of identified peptides were annotated as *Pyrococcus furiosus* sequence when searching the *P. furiosus* proteome against a large database (containing 6,162,582 sequences). In contrast, our study combined results from multiengine database searching of high-resolution fragmentation spectra. Using our approach, 93.4% of peptides identified by a significantly larger database (130,975,891 sequences) matched with those identified by conventional reference database when analyzing the *E. coli* proteome.

Our study represents the first effort to discover gut microbial proteolytic signatures from public datasets, providing a different layer of information beyond taxonomic and protein abundances. The analysis was based on the hypothesis that similar degree of proteolysis should lead to similar relative abundance of semi-tryptic peptides. Using NRASP as an indicator, we observed that microbial semi-tryptic peptides in 447 fecal metaproteomes were enriched in several biological processes including fatty acid, carboxylic acid, glucose, and fucose metabolic processes, BCAA biosynthesis process, protein transport, and bacterial-type flagellum-dependent cell motility, suggesting they underwent more extensive proteolytic regulation. BCAAs (isoleucine, leucine, and valine) are important nutrients in bacterial physiology, and BCAA biosynthesis pathway is essential for optimal growth of many bacteria [45–47]. In this study, we found that NRASP of BCAA biosynthesis process (1.75–2) is higher than that of non-BCAA (~ 1) in fecal metaproteomes. The higher NRASP may offer bacteria more adaptive flexibility in BCAA biosynthesis by proteolysis regulation.

The fecal microbiome serves as a proxy for the gut luminal microbiota but is not fully representative of the mucosa-associated microbiota at the site of disease. Important complementary knowledge could be acquired by systematically characterizing the MLI metaproteomes from different sites, which revealed remarkable location-specific alterations of microbial proteolysis signatures. Interestingly, our findings are consistent with clinical symptoms of IBD within the gastrointestinal tract, where CD mainly occurs in terminal ileum [48] and UC mainly localizes in descending colon and rectum [49]. Accordingly, our results revealed that CD differed from UC and control in terminal ileum and UC differed from the other two groups in descending colon in terms of microbial proteolysis motif and NRASP of biological processes. In terminal ileum, NRASP of transcription, translation, and protein folding decreased in CD while NRASP of translational termination increased. Similarly, decreased NRASP of translation and increased NRASP of translation termination were observed in UC in descending colon. These results potentially indicated the dysregulations of microbial protein synthesis and maturation in IBD.

While we observed several inter-group differences when separately comparing the proteolysis signatures of major taxonomic or functional groups, we could not combine specific taxonomy and function (as what can be performed in metagenomics research) for differential analysis of proteolysis features. This is because of the relatively low proportions (accounted for 15–20% of the total identified peptides) and the high missing values of semi-tryptic peptides across different samples (Additional file 1: Table S1 and Table S3). Currently, the analytical depth of LC-MS-based metaproteomics is still very low compared with metagenomics. Ultra-deep metaproteomics profiling employing on-line or off-line fractionation can provide more in-depth insight into gut microbial proteolysis and may offer the opportunity to combine taxonomy and function in analyzing proteolysis features.

In terms of host factors, we focused on human protease inhibitors and immunoglobulins. Generally, intestinal protease activity increases in IBD and protease inhibition has been proposed as new therapeutic strategy for IBD [50–52]. In our analysis, we found human endogenous protease inhibitors such as Serpins A1 significantly increased in IBD (particularly CD) fecal and MLI samples (Additional file 1: Fig. S7a), probably because the increased protease activity triggers the production of a higher level of protease inhibitors in order to control the destructive nature of protease. There are many host mechanisms that have evolved to regulate host-microbiota interactions, and among these, one of the most widely studied is immunoglobulin A (IgA) [53, 54]. In addition, a recent study has reported that IgG selectively identifies pathobionts in pediatric IBD [55]. Altered microbial composition and metabolic products can trigger mucosal immune responses that mediate IBD [56, 57]. In our study, we did observe different immunoglobulins significantly increased in IBD (Additional file 1: Fig. S7b). Interestingly, microbial proteolysis exhibited negative associations with IgG and IgM but positive associations with IgA. In most cases, we found human immunoglobulins and protease inhibitors were negatively associated with the microbial proteolytic signatures, suggesting the inhibitory effects of these host factors on gut microbial proteolysis events.

Although large-scale data mining of published metagenomics datasets has revealed many new biological insights, this paradigm lags behind in metaproteomics research. Our semi-tryptic peptide centric mining strategy offers a label-free approach to discover signatures of in vivo microbial proteolysis events if experimental conditions are well controlled (e.g., fast sample storage and enzyme inactivation). While results from individual studies can be inconsistent, meta-analysis of multiple published datasets using this approach can increase the confidence of results. On the other hand, abnormally high proportions of semi-tryptic peptides may indicate in vitro proteolysis during sample preparation. Thus, this approach could be used to evaluate and control experiment conditions (e.g., enrichment of microbial cells by differential centrifugation). In addition to gut microbial proteolysis signatures, the approach should also work to explore proteolysis regulations in plant and environmental microbiome if appropriate public or sample-matched sequence database are available.

## Conclusions

Proteolysis regulation is an important strategy for gut microbial adaptation to the fluctuating intestinal environment. Alterations of the gut microbial proteolytic signatures in inflammatory bowel disease are highly diverse and divergent, thus highlighting the need for broader investigations to elucidate their functions. Our data also supports metaproteomics as a valuable approach to investigate the deeper regulation rules of the gut microbiota and host-microbial interactions.

## Supplementary Information

**Additional file 1:** **Table S1.** Microbial semi-tryptic peptides and the cleavage sites in the fecal metaproteomes. **Table S2** Human semi-tryptic peptides and the cleavage sites in the fecal metaproteomes. **Table S3** Microbial semi-tryptic peptides and the cleavage sites in the ascending colon (AsC) MLI metaproteomes. **Table S4** Microbial semi-tryptic peptides and the cleavage sites in the descending colon (DeC) MLI metaproteomes. **Table S5.** Microbial semi-tryptic peptides and the cleavage sites in the terminal ileum (TI) MLI metaproteomes. **Table S6.** Human semi-tryptic peptides and the cleavage sites in the ascending colon (AsC) MLI metaproteomes. **Table S7.** Human semi-tryptic peptides and the cleavage sites in the descending colon (DeC) MLI metaproteomes. **Table S8.** Human semi-tryptic peptides and the cleavage sites in the terminal ileum (TI) MLI metaproteomes. **Table S9-S10.** Human full tryptic peptides in fecal metaproteomes. **Table S11.** Microbial full tryptic peptides in the ascending colon (AC) MLI metaproteomes. **Table S12.** Human full tryptic peptides in the ascending colon (AsC) MLI metaproteomes. **Table S13.** Microbial full tryptic peptides in the descending colon (DeC) MLI metaproteomes. **Table S14.** Human full tryptic peptides in the descending colon (DeC) MLI metaproteomes. **Table 15.** Microbial full tryptic peptides in the terminal ileum (TI) MLI metaproteomes. **Table 16.** Human full tryptic peptides in the terminal ileum (TI) MLI metaproteomes. **Table S17.** NRASP of 20 major taxonomic sub-groups identified in fecal metaproteomes. **Table S18.** NRASP of 35 major biological processes identified in fecal metaproteomes. **Table S19.** NRASP of 32 major enzyme subclasses identified in fecal metaproteomes. **Table S20.** Peptides and altered NRASP. **Table S21.** Peptides identified in the Escherichia coli proteome using reference database and one-step database searching. **Table S22.** NRASP of biological processes identified in Escherichia coli proteome. **Table S23.** Relative abundance of 81 major taxonomic sub-groups identified in fecal metaproteomes. **Table S24.** NRASP of 156 major biological processes identified in fecal metaproteomes. **Table S25.** NRASP of 195 major enzyme sub-subclasses identified in fecal metaproteomes. **Table S26.** Alterations of amino acid frequencies around the cleavage sites in the fecal microbial proteins. **Table S27.** Alterations of amino acid frequencies around the cleavage sites in the fecal human proteins. **Table S28.** NRASP of 57 major biological processes identified in AsC metaproteomes. **Table S29.** NRASP of 56 major biological processes identified in DeC metaproteomes. **Table S30.** NRASP of 51 major biological processes identified in TI metaproteomes. **Table S31.** Alterations of amino acid frequencies around the cleavage sites in the ascending colon (AsC) MLI microbial proteins. **Table S32.** Alterations of amino acid frequencies around the cleavage sites in the decending colon (DeC) MLI microbial proteins. **Table S33.** Alterations of amino acid frequencies around the cleavage sites in the terminal ileum (TI) MLI microbial proteins. **Table S34.** Alterations of amino acid frequencies around the cleavage sites in the ascending colon (AsC) MLI human proteins. **Table S35**. Alterations of amino acid frequencies around the cleavage sites in the decending colon (DeC) MLI human proteins. Table S36 Alterations of amino acid frequencies around the cleavage sites in the terminal ileum (TI) MLI human proteins. Table S36 Alterations of amino acid frequencies around the cleavage sites in the terminal ileum (TI) MLI human proteins. **Fig. S1.** Altered fecal metaproteomes of IBD at different levels revealed by full tryptic peptide based normalized relative abundance. Representative alterations are illustated at different taxonomic levels (a) as well as in different biological processes (b) and enzyme sub-classes (c). Dunn-Bonferroni post-hoc analysis following Kruskal-Wallis test was employed to detect significant difference among three groups (CD, Ctrl, and UC). *P < 0.05 versus Ctrl; **P < 0.01 versus Ctrl; ***P < 0.001 versus Ctrl; ^#^ *P* < 0.05 (CD versus UC); ^##^*P* < 0.01 (CD versus UC); ^###^ *P* < 0.001 (CD versus UC**). Fig. S2.** Principal coordinates analysis (PCoA) based on Bray–Curtis index of semi-tryptic peptide intensity in the fecal metaproteomes. **Fig. S3.** Altered amino acid frequencies around the cleavage sites of human proteins in fecal metaproteomes of IBD. **Fig. S4.** Microbial proteins and human proteins in fecal samples can exhibit similar or reversed alteration trends in certain positions around the cleavage site in IBD. Dunn-Bonferroni post-hoc analysis following Kruskal-Wallis test was employed to detect significant group difference. *P < 0.05 versus Ctrl; **P < 0.01 versus Ctrl; ***P < 0.001 versus Ctrl. **Fig. S5** Hierarchical clustering analysis of altered amino acid frequencies around the cleavage sites of human proteins in MLI metaproteome of IBD. a ascending colon, b descending colon, c terminal ileum. **Fig. S6.** Microbial proteins and human proteins in MLI samples can exhibit similar or reversed alteration trends in certain positions around the cleavage site in IBD. a ascending colon, b descending colon, c terminal ileum. Dunn-Bonferroni post-hoc analysis following Kruskal-Wallis test was employed to detect significant group difference. *P < 0.05 versus Ctrl; **P < 0.01 versus Ctrl; ***P < 0.001 versus Ctrl. **Fig S7.** Increased human protease inhibitors (a) and immunoglobulins (b) in IBD revealed by the label-free quantification (LFQ) intensity

## Data Availability

The mass spectrometry proteomics data are available in the ProteomeXchange Consortium via the PRIDE partner repository with the dataset identifier PXD008675, PXD007819, and PXD000498.

## Abbreviations

BCAA: Branched-chain amino acids
CD: Crohn’s disease
CGR: Culturable Genome Reference
IBD: Inflammatory bowel disease
IGC: Integrated gene catalog
LFQ: Label-free quantification
MLI: Mucosal luminal interface
NRASP: Normalized relative abundance of semi-tryptic peptides
PCA: Principle component analysis
PCoA: Principal coordinate analysis
PEP: Posterior error probability
UC: Ulcerative colitis
